# BLAME-SHIFTING OR SHARING RESPONSIBILITY? CHILDREN’S ROLE IN CHINESE OLDER ADULTS’ END-OF-LIFE MEDICAL DECISIONS

**DOI:** 10.1093/geroni/igae098.1747

**Published:** 2024-12-31

**Authors:** Lu Chen, Wenjun Zhang

**Affiliations:** Renmin University of China, Beijing, Beijing, China (People’s Republic); Renmin University of China, Beijing, Beijing, China (People’s Republic)

## Abstract

Filial piety is the core value shaping intergenerational interactions in China, where children are often considered the proxy decision-makers for older adults’ end-of-life treatment plans. End-of-life care decision-making is a complicated and culturally sensitive process. Prolonging older parents’ lives is often considered a reflection of filial piety in the medical decision-making context, however, budget constraints may weave into the decision-making process. The study addressed the literature gap by empirically investigating the role of children in the end-of-life medical decisions in China. Using data with 579 older decedents who had passed away due to illness from the China Longitudinal Aging Social Survey 2020, we examined the association between role of children, proxied by number of children and relative contribution of children to parents’ medical expenses, and end-of-life decision as a process outcome, measured by a categorical variable with four mutually exclusive groups of whether all life-sustaining medical efforts have been made and whether these decisions imposed financial pressure on their families. Results show that families with fewer children were more likely to pursue all potential treatments and experience financial pressure. Compared to families with shared financial responsibilities by all children, families with shared responsibilities by certain children were more likely to exhaust all potential treatments and face financial strain. Post-hoc analyses further showed that older adults with more children were more likely to be overtreated, particularly among those families with no financial concerns to pursue every potential treatment. Family-level interventions are needed to support end-of-life care decision-making in China.

